# Male fathead minnow transcriptomes and associated chemical analytes in the Milwaukee estuary system

**DOI:** 10.1038/s41597-022-01553-6

**Published:** 2022-08-04

**Authors:** Natàlia Garcia-Reyero, Mark A. Arick, E. Alice Woolard, Mitchell Wilbanks, John E. Mylroie, Kathleen Jensen, Michael Kahl, David Feifarek, Shane Poole, Eric Randolph, Jenna Cavallin, Brett R. Blackwell, Daniel Villeneuve, Gerald T. Ankley, Edward J. Perkins

**Affiliations:** 1 U.S. Army Engineer Research and Development Center, Environmental Laboratory, Vicksburg, MS USA; 2 Institute for Genomics, Biocomputing & Biotechnology (IGBB), Mississippi State University, Starkville, MS USA; 3 Bennet Aerospace, Raleigh, NC USA; 4 U.S. Environmental Protection Agency, Office of Research and Development, Great Lakes Toxicology and Ecology Division, Center for Computational Toxicology and Exposure, Duluth, MN USA

**Keywords:** Gene expression analysis, Environmental monitoring

## Abstract

Contaminants of Emerging Concern (CECs) can be measured in waters across the United States, including the tributaries of the Great Lakes. The extent to which these contaminants affect gene expression in aquatic wildlife is unclear. This dataset presents the full hepatic transcriptomes of laboratory-reared fathead minnows (*Pimephales promelas*) caged at multiple sites within the Milwaukee Estuary Area of Concern and control sites. Following 4 days of *in situ* exposure, liver tissue was removed from males at each site for RNA extraction and sequencing, yielding a total of 116 samples from which libraries were prepared, pooled, and sequenced. For each exposure site, 179 chemical analytes were also assessed. These data were created with the intention of inviting research on possible transcriptomic changes observed in aquatic species exposed to CECs. Access to both full sequencing reads of animal samples as well as water contaminant data across multiple Great Lakes sites will allow others to explore the health of these ecosystems in support of the aims of the Great Lakes Restoration Initiative.

## Abstract (summary)

**Table:** 

Measurement(s)	transcripts • water chemistry
Technology Type(s)	RNAseq • GC/MS
Factor Type(s)	exposure
Sample Characteristic - Organism	Pimephales promelas
Sample Characteristic - Environment	estuary system
Sample Characteristic - Location	Milwaukee, WI, USA

## Background & Summary

The Great Lakes and their tributaries provide significant economic and environmental value to both the United States and Canada, providing 51 million jobs as well as drinking water for 48 million people^1^. However, the levels of complex mixtures, chemical pollutants, and Contaminants of Emerging Concern (CECs) measured in this aquatic ecosystem^2–5^ raise concern for their possible impacts on wildlife health. One such health impact observed in these water systems is the increased rate of fish tumors and deformities, which have an unknown relationship with detected CECs^6,7^.

The Great Lakes Restoration Initiative (GLRI) is a federal program founded in 2010 and led by the US Environmental Protection Agency (USEPA), developed out of a need to protect and restore the Great Lakes fresh water system^8^. At the time of this data collection, GLRI Action Plan II detailed the necessary focus areas for cooperative working groups to achieve the restoration goals of the Initiative^9^. The Toxic Substances and Areas of Concern focus area identified an “Increase [in] knowledge about contaminants in Great Lakes fish and wildlife” as a key objective. We contributed to this task by providing data needed for the development of an improved method for quickly assessing and predicting biological harm.

The data presented here are intended for evaluating the utility of a new predictive toxicology approach (i.e. quickly examining pathways within animals exposed to CECs). The validation of this prediction tool will allow for improved monitoring of biological harm within all Areas of Concern. If assessment of this data reveals CECs to be a non-concern towards wildlife health this information may then be used in part to delist the studied region as an Areas of Concern^10^.

**Fig. 1:**
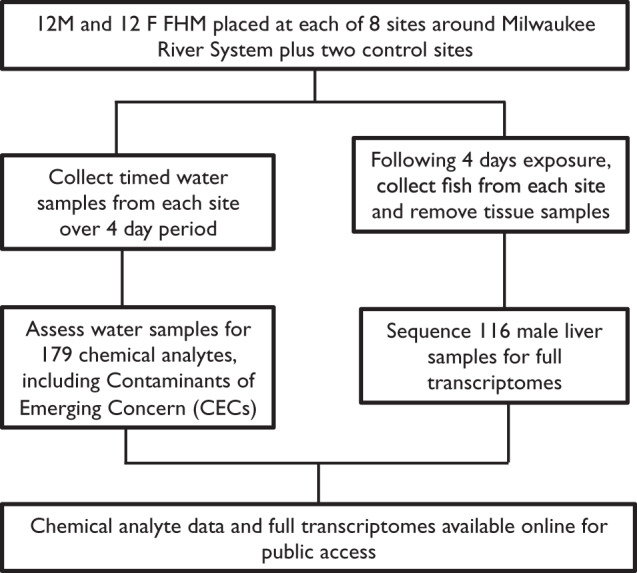
Schematic Overview of the Study Design.

This study addresses the need for increased knowledge about contaminants in Great Lakes wildlife by capturing chemical pollutant information and the associated transcriptomes of exposed aquatic animals (Fig. 1). Caged fathead minnows (*Pimephales promelas*) were deployed across eight exposure sites around the Milwaukee Estuary system and two control sites in June 2017. Following 4-day exposure, male fathead minnows were collected from sites and had liver tissue removed for RNA sequencing. While male and females were included in the study, here we present data from males only. The initial focus on male analysis was due to the potential for endocrine disrupting compounds with estrogenic activity. At these same sites over the same period of exposure, time integrated water samples were collected and assessed for the presence of over 170 relevant chemical analytes. Choice of chemicals was based on two factors. First, a set of wastewater indicators used as a common baseline set for other GLRI integrated study sites was chosen in order to be able to compare the dataset to other sampling sites and years. Secondly, a set of analytes representing polycyclic aromatic hydrocarbons (PAHs) as a major use class of compounds was added. The complete transcriptomes of 116 male fathead minnows as well as chemistry data for multiple Milwaukee River system sites are presented here.

## Methods

### Fathead minnows fish exposures

Fish used in the study were reproductively mature fathead minnows (7–8 months old) from the USEPA Great Lakes Toxicology and Ecology Division (Duluth, MN). All procedures involving animals were reviewed and approved by the Animal Care and Use Committee in accordance with Animal Welfare Act and Interagency Research Animal Committee guidelines. Fathead minnows were shipped on ice, in oxygen-saturated water, overnight to Milwaukee. The study involved four independent shipments of fish, each with its own field control (CON, n = 6 males and 6 females), providing enough animals for deployment at 8 different field sites (n = 12 males and 12 females per site). The CON group fish were held in flow-through conditions using dechlorinated tap water in a controlled laboratory setting at the University of Wisconsin-Milwaukee for the same period that the fish in the field were deployed. An additional set of control fish (n = 12 males and 12 females) were held in flow-through conditions using filtered, UV-treated Lake Superior water for four days at the Great Lakes Toxicology and Ecology Division in Duluth MN (laboratory controls; “GLTED”).

**Fig. 2:**
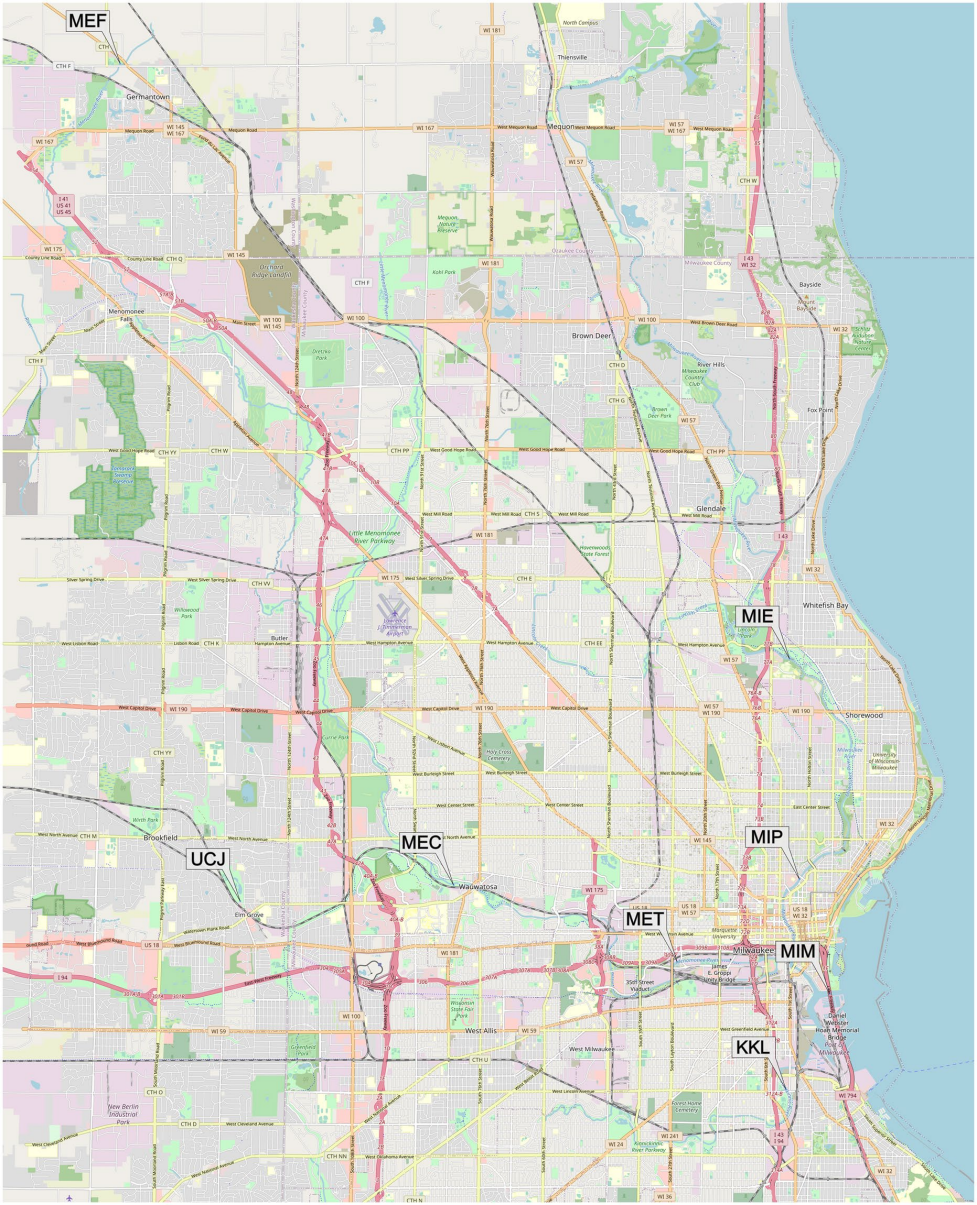
Map of Field Sampling Locations. The site abbreviations correspond to the locations in Online-only Table 1.

Fish for field deployment were driven to the appropriate field location (still in bags of oxygen saturated water), acclimated to the ambient surface water temperature, then deployed in cages as described by Kahl *et al*.^11^ and following a similar approach to Perkins *et al*.^12^. Fathead minnows were caged at each field location, or in the laboratory, for 4 days at eight different sites in or near Milwaukee, Wisconsin (Online-only Table 1, Fig. 2). The locations were: Menomonee River (MET), Milwaukee River at Milwaukee (MIE), Milwaukee River at Mouth at Milwaukee (MIM), Milwaukee River Walnut St at Milwaukee (MIP), Menomonee River near Germantown (MEF), Underwood Creek at Elm Grove (UCJ), Menomonee River at Wauwatosa (MEC), and Kinnicknnic River at Milwaukee (KKL). Two field deployment buoys were anchored to the bottom sediment at each of the sites. Two cages of fish, each containing 6 male and 6 female adult fathead minnows, were attached to buoys, with cages suspended at a depth of 1–2 m. Field controls and GLTED controls were held in 20 L glass aquaria containing 10 L of water. There were six males and six females per tank and fish were held under flow-through conditions with flow rates of approximately 45 ml per minute to each tank. Laboratory-held fish were fed thawed adult brine shrimp, ad libitum, daily. Field caged fish consumed whatever food was available in the water column, but no additional food was provided.

Each fish exposed at a site was considered an independent exposure replicate due to the well-mixed and open nature of the rivers. After 4 days of exposure, all fish from the two cages at each site were transferred into buckets containing surface water collected at the respective site, and transported to a laboratory at the University of Wisconsin-Milwaukee (transit time <60 min) for sampling. Fish were individually anesthetized and euthanized with MS-222 (Argent, Redmond, WA, USA), weighed, and evaluated for any external lesions. Liver tissues were collected from each of the 11–12 males per site and stored at −80 °C until extracted and analyzed. Some sites lost one sample due to animal mortality or sample storage error (MEC, MEF, MIE), yielding 11 total samples from these sites. All fish at site KKL were found dead and not processed for RNA sequencing. Plasma and additional tissues were collected from the exposed males and females for use in other analyses reported elsewhere.

### Water chemistry

At each exposure site, as well as control sites, an automated composite water sampler was attached to the buoy cable, with a water intake hose at the fish level^11^. The autosamplers were programmed to collect water aliquots at 10-min intervals for the entirety of the deployment. The final volume of the 4-day water composite was approximately 10 L. Chemicals extracted from these samples were assumed to be well mixed and representative of the surface water over the period of fish exposure, and so were used as an average measurement of chemicals that caged fish were exposed to across 4 days at that site. Water samples were transferred into precleaned amber glass bottles and shipped overnight on ice to the U.S. Geological Survey (USGS) National Water Quality Laboratory (NWQL) for the analysis of 110 pharmaceuticals (NWQL schedule 2440)^13^ and 69 organic waste compounds (NWQL schedule 4433)^14^. Compounds were extracted using continuous liquid-liquid extraction and methylene chloride solvent, then determined by capillary-column gas chromatography/mass spectrometry^15^. Data are evaluated using the quantitative analysis component of the Agilent MassHunter Workstation software. Specific procedures used (including quality assurance/quality control measures), and complete chemical results for similar Great Lakes Areas of Concern studies are further detailed by the USGS^16^. Concentrations reported as an estimated value were characterized as detected for the different analyses. The principal component analysis (PCA) for the chemical data was done using R [v3.4.4]^17^ and visualized with the ggfortify [v0.4.11] package^18^.

### Sample processing and sequencing

Samples had silica beads added to each tube and processed with mixer mill homogenization before total RNA isolation was conducted using the Nucleospin RNA XS kit following manufacturer’s recommendations. Total RNA samples were measured using a NanoDrop^TM^ 2000 spectrophotometer (NanoDrop Technologies, Wilmington, DE). RNA integrity was assessed using an Agilent 2200 TapeStation (Agilent Technologies, Santa Clara, CA). Libraries were prepared using 125 ng of total RNA per sample using a TruSeq Stranded mRNA LT Sample Preparation Kit (Illumina, San Diego, CA, USA) as per the manufacturer’s instructions. Briefly, the poly-A containing mRNA molecules were purified using magnetic beads, fragmented, and synthesized into first strand cDNA. Next, second strand cDNA was synthesized, a single ‘A’ nucleotide added to the 3′ ends, the single-index adapters ligated, and DNA fragments were enriched to prepare the final libraries. The size and purity of the libraries were determined on the D1000 ScreenTape on the Agilent TapeStation 2200 (Agilent Technologies, Santa Clara, CA, USA). The quantity of the individual libraries was assessed using the KAPA Library Quantification Kit for Illumina Libraries (Kapa Biosystems, Inc., Wilmington, MA, USA) and confirmed using the dsDNA HS Kit on the Qubit 3.0 Fluorometer (Invitrogen, Carlsbad, CA, USA). The concentrations of the libraries were then normalized, pooled together with eight libraries per pool, and quantified using the dsDNA HS Kit on the Qubit 3.0 Fluorometer, followed by further dilution to 5 nM. The pools were then sequenced on a HiSeq. 4000 system (Illumina) at 1 × 150 cycles single-read using the HiSeq. 3000/4000 SBS kit following the manufacturer’s instructions. The raw read quality assessment images were created using quack [v2.0]^19^ and imagemagick [v6.9.10-68]^20^. The multidimensional scaling analysis used the R package tximport [v1.6.0]^21^ to read the counts data, edgeR [v3.20.9]^22^ to run the multidimensional scaling, and ggplot2 [v3.3.3]^23^ to graph the data.

## Data Records

Raw FASTQ files and processed transcript quantification files from the RNA-sequencing of these 116 samples are deposited in NCBI’s Gene Expression Omnibus database, available through the GEO Series accession number GSE144301^24^. Data for positive value chemical analytes obtained for each of these sites is available as a downloadable spreadsheet on Zenodo at 10.5281/zenodo.3608340^25^.

## Technical Validation

### Animal exposures

Two non-exposed, control groups of fathead minnows were held in 20 L glass aquaria filled to 10 L volume under flow-through conditions during the 4-day exposure period: an indoor laboratory facility at USEPA-Duluth (“GLTED”) and an indoor laboratory facility at UW-Milwaukee (“CON”). The GLTED fish were housed in UV-treated, filtered Lake Superior water and CON fish were housed in dechlorinated tap water in a UW-Milwaukee lab.

### Water chemistry

**Fig. 3:**
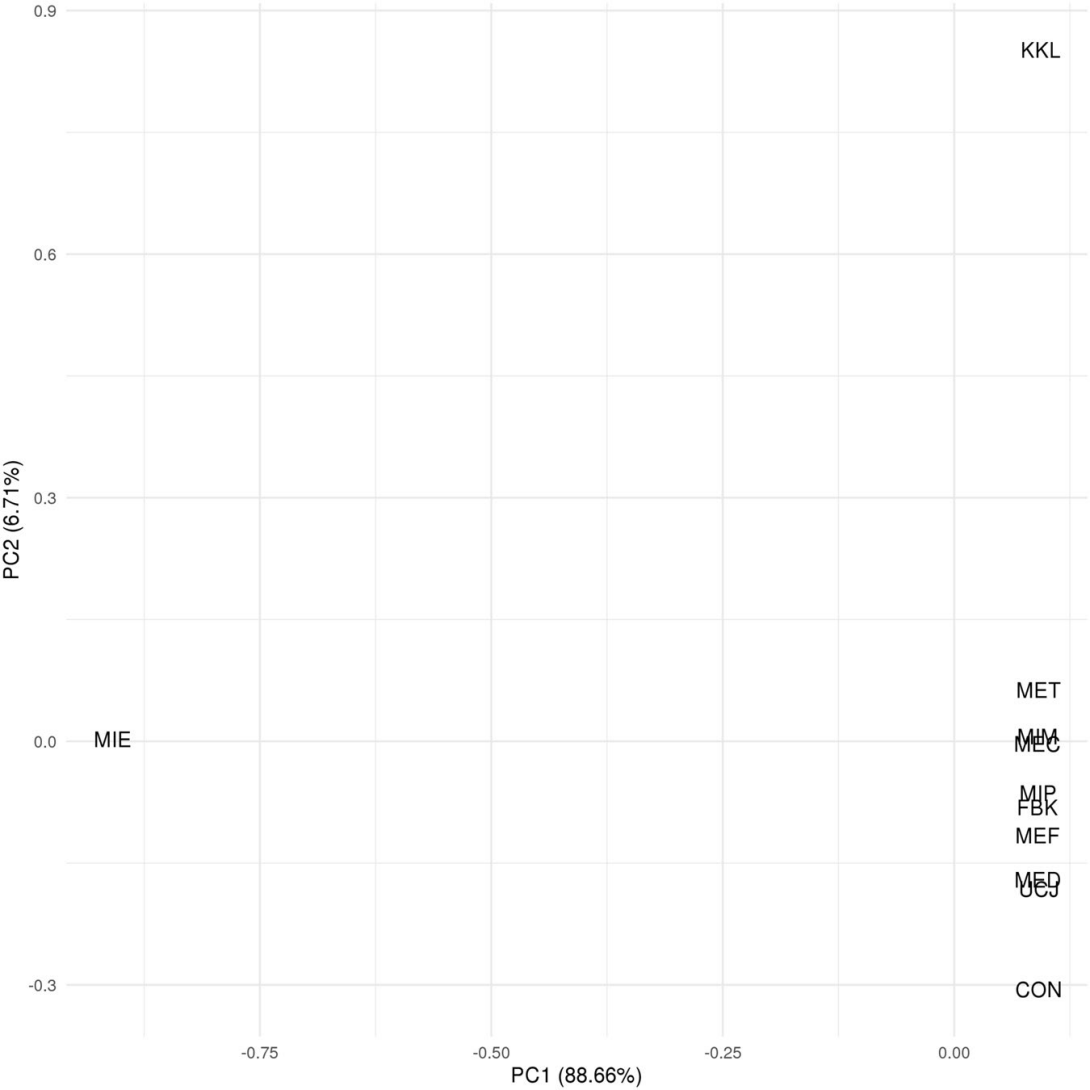
Principal component analysis (PCA) for the chemistry data.

The field blank used for chemical analytical analysis was HPLC-grade water that was pumped through one of the autosamplers similar to those used for the field sites in Milwaukee. This field blank allowed for the detection of any CECs that may have originated from contaminants from the materials used in the autosamplers, or from transport and handling in the field. The principal component analysis (PCA) for the chemical data can be found in Fig. 3. Interestingly, the MIE site seems to be the furthest from the others, and arguably due to the chemical tris(2-butoxyethyl) phosphate. Water chemistry data are available through the web service “Water Quality Portal” that provides access to several U.S. federal water chemistry databases including the USGS National Water Information System (NWIS). To access this data, visit https:// www.waterqualitydata.us, and initiate a query using the USGS station identification numbers, date ranges in online-only Table 1, and choose “Sample results (physical/chemical metadata)”. Alternatively, use the following link for a dynamic query of the database to download the complete data set: https://www.waterqualitydata.us/data/Result/search?siteid*=USGS-04087000;USGS-04087014;USGS-04087098;USGS-04087099;USGS-04087141;USGS-04087170;USGS-04087171;USGS-040870112;USGS-040870855;USGS-040871607&startDateLo=06-07-2017&startDateHi=06-15-2017&mimeType=tsv&zip=no*. (downloaded on 06/07/22 as figshare File 2^26^).

### Transcriptomics

**Fig. 4:**
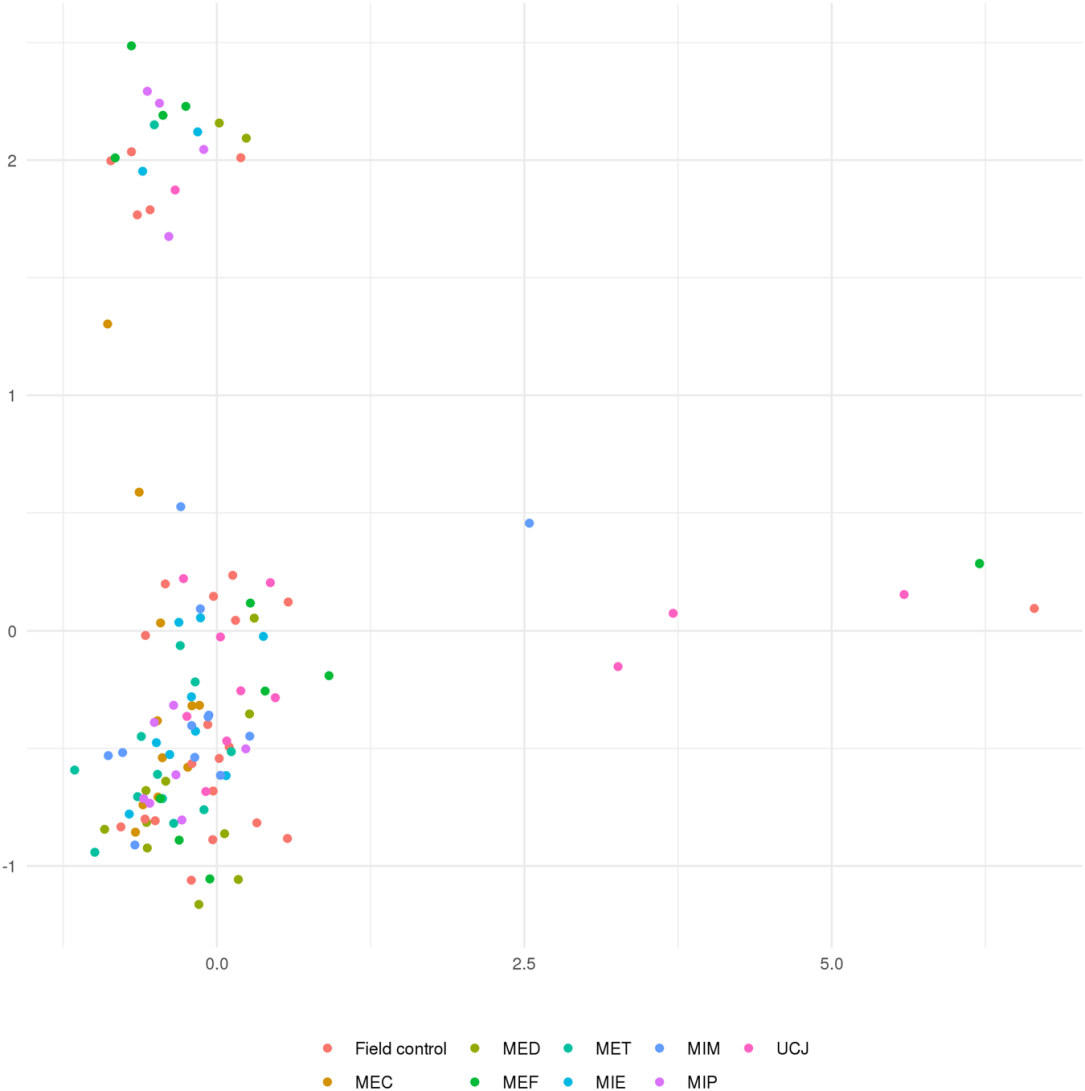
Multidimensional scaling analysis for all transcriptomics samples.

Total RNA samples were measured using a NanoDrop^TM^ 2000 spectrophotometer (NanoDrop Technologies, Wilmington, DE). RNA integrity was assessed using an Agilent 2200 TapeStation (Agilent Technologies, Santa Clara, CA). An RNA integrity number >8.0 from the Agilent 2200 TapeStation was used as criteria for acceptable RNA quality. No negative controls nor spike-in controls were used. The custom reference transcriptome used was aligned using NCBI BLAST (version 2.6.0+) against the fathead minnow and zebrafish mRNA sequences in Genbank for annotation purposes. The raw read quality assessment images can be found in figshare File 3^26^. The multidimensional scaling analysis for all samples is shown in Fig. 4.

## Usage Notes

These two sets of independent data may be analyzed separately or used to together for exploration of associations between fathead minnow genome changes and water chemistry. Site-specific genomic changes found in samples may be associated with contaminant exposures. For example, to assess tumorigenicity, genes associated with tumor development may be identified and examined within fish transcriptomes for differences between exposure sites and controls. If differences are present, follow-up analysis may involve examining chemical analyte composition differences between sites. Fish transcriptomes provided here may also be useful for comparison to other similar studies. Chemistry data alone may be used for predictive risk assessment, and could also be analyzed in conjunction with site-specific information, such as proximity to city center, or biodiversity of immediate area.

Lab control animals were fed thawed adult brine shrimp while field-exposed animals freely fed from the water column. It is uncertain what impact, if any, these differences in diet had on the observed transcriptomic changes. No differences in fish weight were observed between sites and all fish appeared to have been eating, which suggests equal access to food between the exposure and control groups. A few polycyclic aromatic hydrocarbons (PAHs) were detected at low concentrations in CON water (see figshare File 1^25,26^).

For all sites, chemical analyte data are reported such that a non-detect (ND) is assigned to any chemical value measured at ½ the detection limit. If there is an estimated value measured below detection, ¼ the lowest estimated value is reported. No predictions about the quality of water between field sites and controls were generated at the time of sample collection. This unbiased sampling and presentation of data is thus given without any expectation of site-specific trends. Complete USGS water quality data are downloadable from USGS National Water Information System (NWIS) 10.5066/F7P55KJN and figshare File 1^26^.

It is worth noting that this dataset has some limitations that should be taken into account. As with most field exposures, finding appropriate controls is always challenging. Here we report two sets of controls, and their differences with the field-exposed fish should be noted. Caged fish are eating different food than control fish, which could lead to variable exposure to contaminants. Stress levels might be different in caged versus control fish, which could affect physiological responses. As chemistry is targeted and measures a specific set of compounds, other chemical stressors might be missing from our analysis. We believe that this dataset can help inform future field experiments and particularly experimental design and conclusions.

## Data Availability

No custom code was used to generate or process the data presented in this manuscript.

## Online-only Table

**Online-only Table 1:** Site names, abbreviations, geolocation, and date of sampling.

Site	Abbreviation	Latitude	Longitude	Dates	Final No. Male FHM Liver Samples
Menomonee River at 25^th^ St., at Milwaukee, WI	MET	43.032	−87.946	08–12 June 2017	12
Milwaukee at Estabrook, at Milwaukee, WI	MIE	43.101	−87.911	10–14 June 2017	11
Milwaukee River at Mouth at Milwaukee, WI	MIM	43.025	−87.899	11–15 June 2017	12
Milwaukee River Walnut St at Milwaukee, WI	MIP	43.051	−87.908	11–15 June 2017	11
Menomonee River at Freistadt Rd near Germantown, WI	MEF	43.236	−88.119	10–14 June 2017	11
Underwood Creek at Juneau Blvd at Elm Grove, WI	UCJ	43.046	−88.081	09–13 June 2017	12
Menomonee River near Church St at Wauwatosa, WI	MEC	43.050	−88.015	09–13 June 2017	11
Kinnickinnic River at Lincoln Ave at Milwaukee, WI	KKL	43.003	−87.912	08–12 June 2017	0^1^
USEPA GLTED Lab at Duluth, MN	GLTED	N/A	N/A	16–20 June 2017	12
Control at UW-Milwaukee (Field Controls)	CON	N/A	N/A	08–15 June 2017 (FHM exposure); 10–14 June 2017 (water sampled)	24
Milwaukee River at Mouth at Milwaukee, WI (Field Blank)	FB	N/A	N/A	11–15 June 2017	N/A; Water chemistry data only

Eleven to twelve male liver tissues were collected and analyzed from adult male fathead minnows caged at sites within the Milwaukee estuary. Twelve samples were taken from control site GLTED, as well as another 24 samples taken from the field control site at UW-Milwaukee. Site FBK was used as a field blank for water chemistry control collection.

^1^No samples were obtained from this site because of mortality.
